# Inhibition of neddylation regulates dendritic cell functions via Deptor accumulation driven mTOR inactivation

**DOI:** 10.18632/oncotarget.9543

**Published:** 2016-05-21

**Authors:** Mengmeng Cheng, Shurong Hu, Zhengting Wang, Yaofei Pei, Rong Fan, Xiqiang Liu, Lei Wang, Jie Zhou, Sichang Zheng, Tianyu Zhang, Yun Lin, Maochen Zhang, Ran Tao, Jie Zhong

**Affiliations:** ^1^ Department of Gastroenterology, Ruijin Hospital, Shanghai Jiao Tong University School of Medicine, Shanghai, China; ^2^ Department of Surgery, Ruijin Hospital, Shanghai Jiao Tong University School of Medicine, Shanghai, China; ^3^ Department of Hepatobiliary-Pancreatic Surgery, Zhejiang Provincial People's Hospital, Hangzhou, China

**Keywords:** neddylation, dendritic cell, mTOR, Deptor, inflammatory bowel disease, Immunology and Microbiology Section, Immune response, Immunity

## Abstract

Neddylation, a newly identified post-translational modification, is significant for the activity and stability of target proteins. The exact role of neddylation in the pathogenesis of inflammatory bowel disease, specifically those mediated by dendritic cells (DCs), was still rarely reported. Here, we showed that inhibition of neddylation protected mice from mucosal inflammation. Targeting neddylation also inhibited DC maturation characterized by reduced cytokine production, down-regulated costimulatory molecules and suppressed capacity in allogeneic T cell stimulation. Additionally, inactivation of neddylation promotes caspase dependent apoptosis of DCs. These phenomena were attributed to the inactivation of mTOR, which was caused by Cullin-1 deneddylation induced Deptor accumulation. Together, our findings revealed that neddylation inhibition suppressed DC functions through mTOR signaling pathway and provided a potential therapeutic opportunity in inflammatory bowel diseases.

## INTRODUCTION

Inflammatory bowel disease (IBD) is a chronic relapsing gastrointestinal inflammatory disease with an accelerating incidence [1, 2]. A great deal of scientific research revealed that IBD was accompanied with abnormal immune regulation, exaggerated mucosal inflammation in response to constituents of the intestinal flora in genetically predisposed individuals [3, 4]. As the most powerful antigen-presenting cells, dendritic cells (DCs) could recognize microbial structures in intestine [5–8]. During this process, DCs up-regulated their microbial recognition receptors, increased pathologically relevant cytokine production, promoted costimulatory molecules expression and strengthened ability in T cell activation, resulting in intestinal inflammation and inappropriate immune response that characterized IBD [9–11]. Given the unique role of DCs in immune response and the relevance between IBD and immune disorders, clarifying the regulatory factors of DC in the progression of IBD might contributed to the mechanistic understanding of this complex disease.

Neddylation, a newly identified type of protein post-translational modifications, is a process that mediated by a set of enzymes and covalently modifies target proteins with Nedd8 (neural precursor cell expressed, developmentally down-regulated 8) [12, 13]. Conjugation of Nedd8 to Cullins could dramatically raise the activities of Skp-Cullin-F box proteins (SCF) E3 ubiquitin ligase, which could assemble ubiquitin to a series of proteins and targeting them for proteasomal degradation [14–17]. More specifically, neddylation does not mediate the polyubiquitination of substrates for degradation directly, whereas, it might probably regulates cellular functions *via* influencing the stability and activity of target proteins.

Previous studies of neddylation were mainly focused on cancer therapy, based on the findings that neddylation inhibitor suppressed the growth of diverse cancer cell lines [18–21]. Recently, it was discovered that Cullin neddylation status influenced by bacterial products could cause epithelial signaling changes [22, 23], indicating a possible relevance between neddylation and mucosal inflammation. Furthermore, inhibition of neddylation was reported to repressed NF-κB mediated proinflammatory cytokine production in macrophages and DCs [24, 25], which suggested neddylation might be also involved in immune regulation. Given that IBD is characterized by exaggerated intestinal inflammation and immune dysregulation that initiated by DCs, we proposed that neddylation might has effect on DCs mediated IBD pathogenesis, which still needed direct biological and mechanistic evidences.

Herein, we defined the role of neddylation in regulating DCs functions, by using a small molecule inhibitor of neddylation, MLN4924. We found that MLN4924 showed a therapeutic efficiency on murine IBD model *in vivo* and suppressed DCs maturation *in vitro via* inactivating mTOR signaling pathway, which provide a new opportunity on IBD therapy.

## RESULTS

### Neddylation inhibitor MLN4924 protects mice from clinical signs of colitis

**Figure 1:**
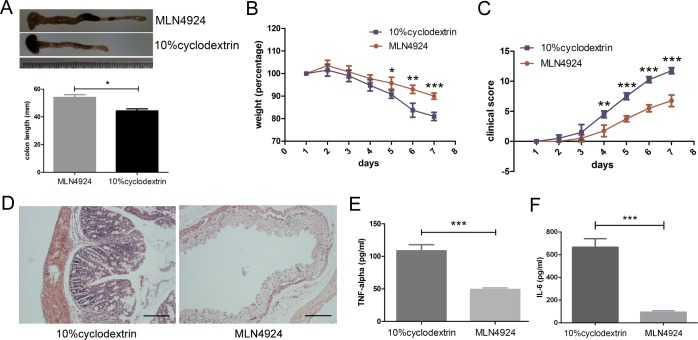
Neddylation inhibitor MLN4924 attenuates DSS-induced colitis in mice **A.** MLN4924 prevented colon shortening in DSS-induced colitis. **B.** Weight loss curve for MLN4924 or cyclodextrin treated DSS mice. **C.** Clinical score represented colitis severity. **D.** Representative micrograph showed attenuated inflammation in MLN4924 treatment group compared with control group. Scale bar for 200μm. **E.**-**F.** MLN4924 reduced cytokine secretion measured by ELISA. Results were presented as the mean ± SEM. *p < .05, **p < .01, ***p < .001.

In order to confirm whether neddylation had any effect on inflammatory injury and autoimmune disorders, we examined the *in vivo* effect of a neddylation inhibitor, MLN4924, in an IBD model. Mice received 4% DSS (dextran sulfate sodium) were divided into two groups, given either 30mg/kg MLN4924 or 10% cyclodextrin intraperitoneal injection daily, respectively. The length of colon from control group was shorter than MLN4924 treated group and the stools of control mice were red and shapeless (Figure 1A), suggesting that MLN4924 ameliorated the DSS-induced colon shortening. Moreover, mice treated with MLN4924 lost weight in a relatively moderate way in response to DSS administration compared with the control group (Figure 1B). Additionally, a reduction of clinical scores was observed in MLN4924 treated group (Figure 1C). These results indicated that neddylation inhibition alleviated colitis development at a certain extent.

### MLN4924 treatment mitigates colon inflammation

Histological analysis of colon tissue sections from mice treated with MLN4924 showed little inflammatory foci, whereas inflammation was observed in the cyclodextrin group as reflected in intestinal tract thickening, inflammatory cell infiltration and goblet cell aggregation (Figure 1D). Thus, MLN4924 remarkably attenuated colon inflammation in murine colitis. Mice from MLN4924 treated group also generated a lower serum level of TNF-α and IL-6 in serum, which represent the acute inflammatory response, compared with the controls (Figure 1E-1F), indicating that inhibition of neddylation had effect on mucosal inflammation therapy.

### Inhibition of neddylation exhibits decreased LPS-induced proinflammatory cytokines secretion in DCs

**Figure 2:**
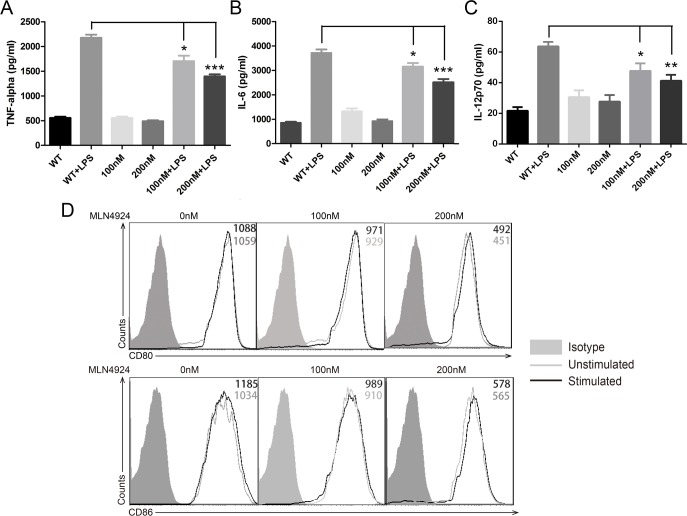
Inhibition of neddylation suppressed DC functions ELISA quantification of **A.** TNF-α, **B.** IL-6 and **C.** IL-12p70 release in the presence or absence of vehicle, MLN4924 and LPS in BMDCs at indicated doses. **D.** CD80 (upper) and CD86 (lower) surface marker expression levels were declined with the risen dosage of MLN4924. One representative experiment of 3 is shown in cells treated in triplicate. *p < .05, **p < .01, ***p < .001.

Since inflammatory injury and innate immunity play a more important role than adaptive immunity in this IBD model. We thought of the possible involvement of DCs, which were crucial in the development of DSS-induced colitis and act as a bridge between innate and adaptive immunity, rather than adaptive immune cells like T cells and B cells [11, 26]. Therefore, we evaluated the release of proinflammatory cytokines in the supernatants of DCs in the exposure of MLN4924 by the means of ELISA. However, we examined no significant difference in the secretion of cytokines when dendritic cells were treated with MLN4924 compared with the control group. Interestingly, the up-regulation of TNF-α and IL-6 was dramatically inhibited by MLN4924 when cells were stimulated with LPS and it was in a dose dependent manner (Figure 2A-2B). These results indicated that MLN4924, a neddylation inhibitor, could reduce LPS-induced secretion of proinflammatory cytokines in DCs, which was in accordance with the observations by Mathewson [24]. We also observed reduced secretion of IL-12p70, a key cytokine produced by DCs for Th1 (T helper 1) cells development, suggesting restricted capacity of DCs for T cell activation (Figure 2C). However, the distinctive role of neddylation in this process remained elusive.

### Costimulatory molecules of DCs are down-regulated by neddylation inhibitor

Having demonstrated reduced LPS-induced cytokines production with MLN4924 treatment, we next evaluated phenotypes of DCs. BMDCs (bone marrow-derived dendritic cells) were cultured in graded concentration of MLN4924 with the stimulation of GM-CSF and IL-4. Phenotype analysis was done at day 6. The expression level of activation markers CD80 and CD86 were down-regulated by MLN4924 treatment, and it was dose dependent (Figure 2D). Since up-regulated costimulatory molecules were used as standard biomarkers to determine DC activation status, these results indicated that neddylation blockade impaired the activation of DCs, in addition to reduced cytokines secretion, thus inhibited the maturation of DCs.

### Stimulate activity of DC is reduced in the presence of MLN4924

**Figure 3:**
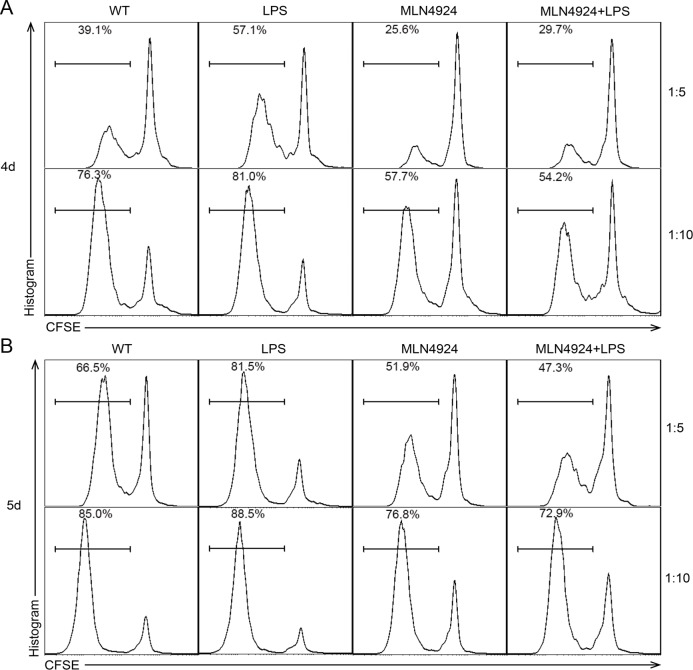
Stimulate activity of DC is reduced in the presence of MLN4924 BALB/c BMDCs were pretreated with or without indicated dose of MLN4924. After isolation and irradiation, CD11c^+^ DCs were used as stimulators of CD4^+^ T cells and co-cultured for **A.** 4 days and **B.** 5 days at the ratio of 1:5 and 1:10. The proliferation rate of T cells was reduced with the presence of MLN4924, which means that inhibition of neddylation could decline the stimulate activity of DCs.

Then we analyzed the stimulate ability of DCs on allogeneic T cells to gain a better understanding of neddylation process during DC maturation. BALB/c BMDCs were pretreated with indicated dose of MLN4924 and co-cultured with C57BL/6 CD4^+^ T cells after irradiation. In the presence of neddylation inhibitor, DCs displayed a reduced stimulating capacity in the proliferation of CD4^+^ T cells, as determined by MLR (mixed leukocyte reaction) at both 4 days (Figure 3A) and 5 days (Figure 3B). This result indicated that MLN4924 treatment weakened the ability of DCs in stimulating allogeneic T cells proliferation. In summary, MLN4924 treatment showed a protective effect on inflammation, characterized by reduced cytokine production during activation, down-regulated costimulaory molecules expression during maturation and impaired stimulatory capacity of DCs.

### Blockade of neddylation impairs CD4^+^ T cell proliferation

**Figure 4:**
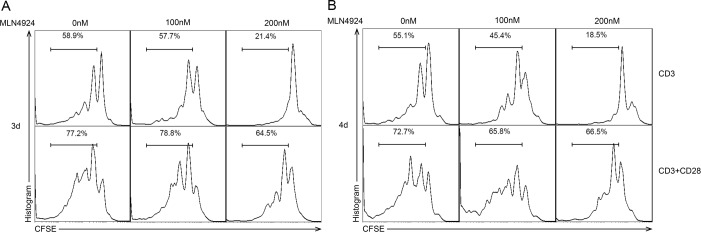
Blockade of neddylation impairs CD4^+^ T cell proliferation CFSE-labeled CD4^+^ T cells were stimulated with anti-CD3/CD28 mAbs for **A.** 3 days and **B.** 4 days in graded dose of MLN4924 exposure, after which cell division was analyzed by flow cytometry. The results suggested that MLN4924 suppressed CD4^+^ T cell proliferation.

To verify whether the blockade of neddylation would have any effect directly on T cells apart from impairing the activity of DCs on T cell stimulation, we isolated and stimulated CD4^+^ T cells with anti-CD3 and anti-CD28 functional antibodies for 72 hours (Figure 4A) and 96 hours (Figure 4B) in different concentrations of MLN4924. Interestingly, MLN4924 significantly suppressed T cell proliferation, suggesting that neddylation inhibitor could influence T cell functions both directly and indirectly, which need further studies to investigate.

### MLN4924 induces apoptosis of BMDCs

**Figure 5:**
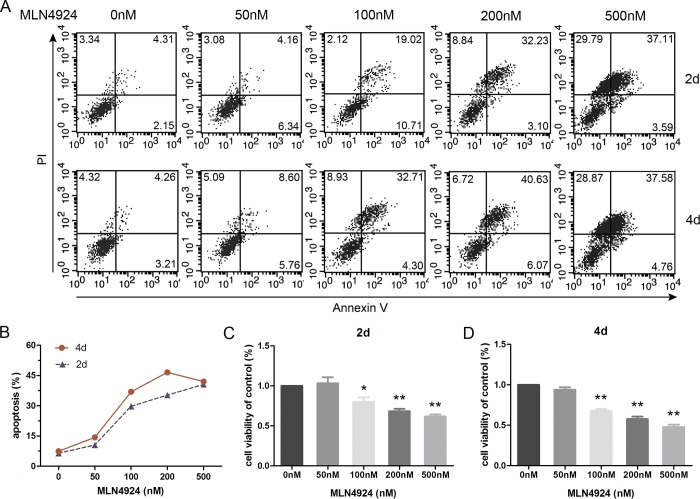
Inhibition of neddylation induces apoptosis of BMDCs BMDCs were cultured with different doses of MLN4924 for 2 or 4 days. **A.** Cells were stained with Annexin V to analysis the impact of concentration and time on apoptosis. **B.** The curves represent the percentage of apoptosis following 2 and 4 days treatment. Trypan blue exclusion assay was employed to evaluate cell viability in 2 days **C.** and 4 days **D.** treatment.

Given the fact that neddylation inhibition suppressed DC maturation, we tried to discover additional functional modulation on DCs and performed apoptosis assay. We found that low dose of inhibitor had little effect on DCs and make a similar behavior to spontaneous apoptosis, whereas medium concentrations induced DC apoptosis in a time dependent manner (Figure 5A-5B). Additionally, we observed a slight decline of apoptosis rate in high dose MLN4924 treatment group compared with the medium dose group by 4 days treatment, which was probably correlated with more dendritic cell deaths. We also used trypan blue exclusion assay and found that DC viability was inhibited by the blockade of neddylation in a dose and time dependent manner (Figure 5C-5D). Thus, MLN4924 enhanced DC apoptosis.

### Neddylation inhibition induces caspase dependent apoptosis

**Figure 6:**
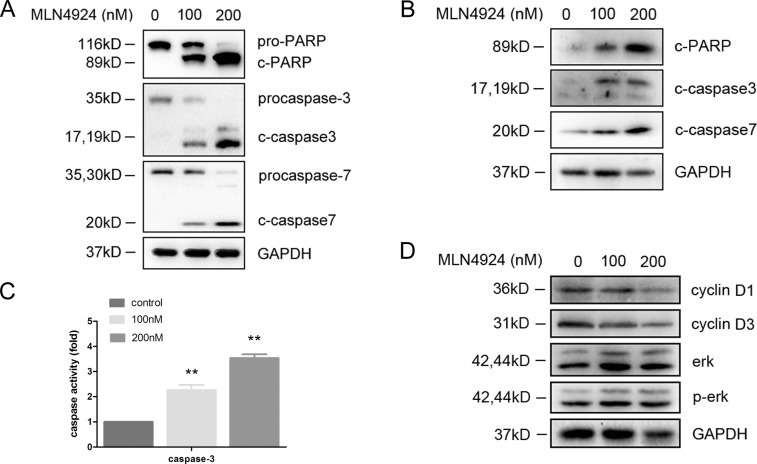
MLN4924 induced apoptosis is caspase dependent BMDCs were cultured with MLN4924. Total cellular protein extracts were prepared and subjected to immunoblotting by using **A.** anti-PARP, anti-caspase-3, anti-caspase-7 antibodies and **B.** anti-cleaved PARP, anti-cleaved-caspase-3, anti-cleaved-caspase-7 antibodies. **C.** Caspase-3 activity was examined by caspase activity kit. **D.** Immunoblotting analysis was performed for erk, p-erk, cyclin D1 and cyclin D3 expression.

In order to further understand the mechanism of apoptosis induction, expression levels of caspases in DCs were examined by immunoblotting in the presence of MLN4924. Cleaved caspase-3, -7 and cleaved PARP expressions were induced by MLN4924 treatment in a dose dependent manner (Figure 6A-6B). We also detected caspase-3 activation and found that the activity of caspase-3 was significantly increased following MLN4924 treatment (Figure 6C). This further supported that inactivation of neddylation could induce DC apoptosis and it was caspase dependent.

### Cell cycle regulation is related with neddylation inhibition

Since MAPK/ERK signaling pathway also plays a role in regulating apoptosis [27, 28], the effect of neddylation on erk activation in DCs was examined. However, no significant change was observed in the level of erk or p-erk by MLN4924 treatment, suggesting that inhibition of neddylation induced apoptosis in DC was independent of MAPK/ERK pathway (Figure 6D). Considering the close relationship between cell cycle and apoptosis, we also investigated the expressions of cyclins in order to determine whether cell cycle regulation was involved. Interestingly, cyclin D1 and D3 protein levels were decreased by MLN4924 treatment, indicating that neddylation suppression induced apoptosis would be cell cycle regulation related, which need further studies to explore (Figure 6D).

### MLN4924 inhibits mTOR activity

**Figure 7:**
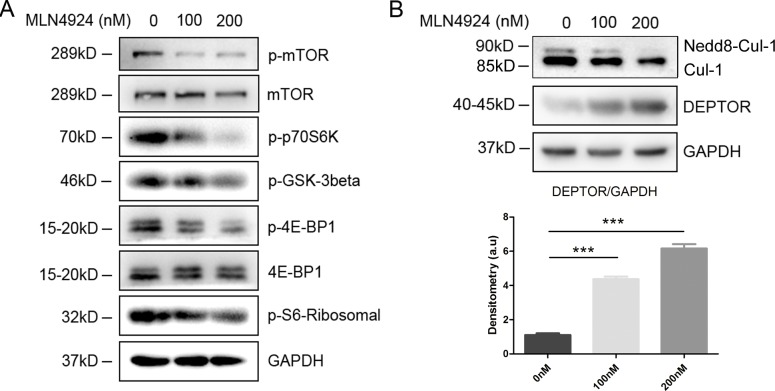
Inhibition of neddylation triggers Deptor accumulation to inactivate mTOR signaling pathway **A.** Cell lysates of the CD11c^+^ DCs sorted from the MLN4924 treated BMDCs were separated by SDS/PAGE and immunoblotted with the indicated antibodies representing mTOR signaling pathway. **B.** Cell lysates of the CD11c^+^ DCs were subjected to immunoblotting with anti-Deptor and anti-Cul-1 antibody (upper), and signal intensity was analyzed (lower).

mTOR was reported to be a well-established regulator of DC functions and downstream signaling [29–31]. For further mechanistic investigations, we found that MLN4924 treatment resulted in a remarkable decreased phosphorylation of mTOR, and reduced mTOR activity, as reflected by dose dependent inhibition of p70S6K and 4E-BP1 phosphorylation (Figure 7A). Furthermore, a similar observation, but to a lesser extent, revealed that MLN4924 treatment led to reduced phosphorylation of S6-Ribosomal and GSK-3β. These results indicated that neddylation inhitor could suppress DC functions, at least partly if not all, *via* mTOR signaling pathway.

### Inhibition of neddylation leads to Deptor accumulation

Next, we tried to discuss the underlying mechanisms through which MLN4924 decreased mTOR activity. It was reported that Deptor, a well-established negative regulator of mTOR kinase, could be recognized by SCF ubiquitin ligase for polyubiquitination and degradation [32, 33]. Furthermore, SCF ubiquitin ligase activity could be raised by the conjugation of Nedd8 to Cullin-1 (Cul-1) [14], indicating inhibitory effect of deneddylated Cul-1 on Deptor degradation. Therefore, we assessed the protein level of Deptor in different concentrations of MLN4924 by immunoblotting assay. As the result, Cul-1 neddylation was inhibited, reflected as a decrease in Nedd8-Cul-1 band, which was accompanied with Deptor accumulation in the presence of MLN4924 (Figure 7B). Thus, we concluded from these results that blockade of neddylation have inhibitory effects on DCs, which were induced by Deptor accumulation driven mTOR signaling inactivation.

## DISCUSSION

In this study, we investigated the inhibitory effect of deneddylation on DC maturation and mucosal inflammation. More specifically, these phenomena included defective cytokine secretion, reduced costimulatory molecules expression, suppressed capacity in allogeneic T cell stimulation and murine colitis alleviation. In addition, we showed that neddylation inhibition induced caspase dependent apoptosis in DCs and put forward that deneddylation of Cul-1 driven Deptor accumulation and following mTOR inactivation was responsible for these phenomena. In summary, our results identified neddylation as a pivotal process in DC mediated IBD pathogenesis and an effective inflammation regulator.

Previous studies had shown that IBD was correlated with neddylation status in epithelial cells, which could be influenced by diversity bacterial products in intestine [22, 23, 34]. However, limited information was available regarding the direct role of neddylation in IBD pathogenesis, specifically those mediated by DCs. Mathewson et al. demonstrated that MLN4924 repressed NF-κB mediated proinflammatory cytokines production by DCs in response to both infectious and noninfectious stimuli [24]. Their work, however, was mainly focused on defective cytokine production, whereas the present study addressed other functions, such as differentiation, maturation and apoptosis, that regulated by neddylation.

To address the role of neddylation in DC functions, we initially evaluated the cytokine production in neddylation inhibition. MLN4924 repressed LPS-induced TNF-α and IL-6 production, which was consistent with the result of Mathewson et al. We also observed reduced secretion of IL-12p70, a key cytokine produced by DCs for Th1 differentiation, suggesting restricted capacity in T cell activation and immune response development. DCs showed down-regulated expression of costimulatory molecules and weakened ability in stimulation, implying that MLN4924 impeded DC maturation. Moreover, MLN4924 attenuated DSS-induced murine colitis, indicating that inhibition of neddylation attenuated inflammation *in vivo*. Taken together, our results extended previously reported findings to a murine model of IBD and other DCs functions, hinting at potential beneficial effect of MLN4924 in various inflammatory diseases and emphasizing the importance of neddylation in DC maturation.

We also observed caspase dependent apoptosis in DCs by neddylation inhibition, suggesting that MLN4924 not only suppressed the maturation from immature precursors but also induced apoptosis of the end-stage mature DC, resulting in the reduction of functional DCs. Furthermore, caspase-3 was reported to restrict the maturation and expression of peptide-loaded major histocompatibility complex class II molecules of DCs [35], despite its classical role as a positive regulator of apoptosis. These literatures provided evidences for the relationship between maturation and apoptosis of DCs. Growing evidence suggested that apoptosis was frequently associated with cell cycle progression [36, 37]. Arrest in late G_1_ or S phase could accelerate apoptosis, moreover, arrest DCs in G_0_ phase made DCs unable to stimulate allogeneic T cells [38]. Accordingly, we found down-regulated protein level of cyclin-D1 and cyclin-D3 in MLN4924 treated DCs. These observations indicated that cell cycle regulation might participate in the neddylation progress of DCs, while clearer evidences were still needed to be explored.

**Figure 8:**
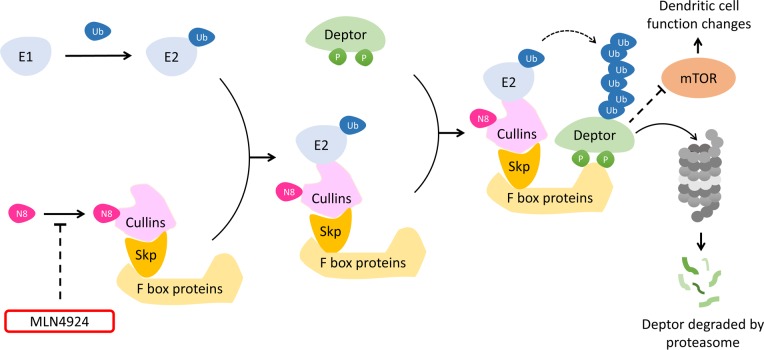
A model for the role of MLN4924 in mTOR signaling inactivation Neddylation inhibitor MLN4924 suppressed the conjugation of Nedd8 to Cullins and reduced SCF mediated polyubiquitination of Deptor for proteasomal degradation. The accumulation of Deptor inactivated mTOR signaling pathway, resulting in dendritic cell biological functions suppression.

In terms of mechanism, the accumulation of Deptor, an inhibitory protein of mTOR, was uncovered as a distinct medium for MLN4924 induced inhibitory effects on DCs. Our present work revealed that MLN4924 could suppress the conjugation of Nedd8 to Cul-1 and thus weakened its ability in ubiquitin-mediated Deptor degradation. These results indicated that the functional suppression of DCs was mainly induced by Cul-1 deneddylation triggered Deptor accumulation and the following mTOR inactivation (Figure 8). Meanwhile, it was reported that NF-κB signaling is also involved in cytokine production changes in DCs in response to neddylation inhibition [24]. Furthermore, several other substrates, which could also be modified by Nedd8, had been identified, in addition to the currently reported Cul-1 [39–41]. Much more interesting, some of these substrates, such as Cullin-2 and HIF-1α (hypoxia-inducible factor-1α), could also influence DCs activation and maturation directly or indirectly [42–44]. On the whole, neddylation inhibition might suppress DC functions *via* modulating multiple signaling pathways in a neddylation dependent manner according to diverse Nedd8 conjugative proteins. Further studies are still needed for evaluating the effect of these proteins on DCs functional regulation.

In conclusion, these experiments validated that neddylation inhibtion could suppress DC functions. Cul-1 deneddylation led to Deptor accumulation and subsequent mTOR inactivation, which had an inhibitory effect on DCs. Strategies targeting neddylation to suppress dendritic cell function might be viable therapeutic intervention against diseases associated with immune dysfunction.

## MATERIALS AND METHODS

### Regents and antibodies

MLN4924 was purchased from Med Chem Express. Antibodies (Abs) for immunoblotting were all obtained from Cell Signaling Technology, except Deptor (Proteintech Group), Cullin-1 (Abcam, Cambridge, MA), cyclin-D1 and cyclin-D3 (Beyotime Institute of Biotechnology). The HRP-linked secondary antibodies were purchased from Sigma-Aldrich. Antibodies for APC-CD11c, PE-CD80, PE-CD86 and FITC-CD4 were purchased from eBioscience. Carboxyfluorescein diacetate succinimidyl ester (CFSE) was obtained from Invitrogen.

### Clinical parameters measuring and histology

Male 6-8 week old C57/BL6 mice were purchased from Shanghai laboratory animal center and maintained in the specific pathogen-free central animal facility of Ruijin hospital affiliated to Shanghai Jiaotong University. The mice were administrated with DSS (4% wt/vol; 36-50kDa; MP Biomedicals) continuously for 7 days to induce colitis. Either 30mg/kg MLN4924 or 10% cyclodextrin (Sigma-Aldrich) intraperitoneal injection was given every day. Stool consistency, occult blood and weight loss were recorded daily to calculate clinical scores. Colon sections (distal to cecum) were cut and fixed in 10% buffered formalin, paraffin embedded, cut into 3 to 5 μm sections, and stained with hematoxylin/eosin.

### ELISA

Supernatants from cultured dendritic cells on day 6 and murine serums were collected and frozen. TNF-α, IL-6 and IL-12p70 concentrations were quantified according to the manufacturer's instructions. Samples were run in triplicate using the ELISA Ready-SET-Go! Kit (eBioscience). Sensitivities for TNF-α, IL-6 and IL-12p70 were 8, 4 and 15pg/ml, respectively. Detection ranges were TNF-α: ~1000pg/ml; IL-6: ~500pg/ml and IL-12p70: ~2000pg/ml. Inter and intra-variations were less than 11.5% for all cytokines.

### Generation and phenotype analysis of bone marrow-derived dendritic cells

Single cell suspension from bone marrow of C57BL/6 mice was got after red blood cell lysis. Cells were stimulated with 10ng/ml GM-CSF and IL-4 for DC differentiation and maturation. Indicated concentrations of MLN4924 were added on day 2 of culture and the supernatant was replaced with fresh cytokine-containing medium (with or without MLN4924) every two days. On day 6, APC- and PE- Abs were used to detect the expression of CD11c, CD80 and CD86. Data was acquired with a flow cytometer and analyzed using FlowJo 6.8 (Tree Star) software packages.

### Mixed leukocyte reaction

BMDCs of BALB/c mice were generated with or without the exposure of MLN4924 as described before. Allogeneic CD4^+^ T cells were purified from murine spleen and lymph nodes using CD4^+^ T cell isolation kit (Meltenyi biotech), and were labeled with CFSE. After γ-irradiation (20 gray), DCs (1 × 10^5^/ml) were cultured with labeled CD4^+^ T cells in a 4 days and 5 days MLR at the ratio of 1:5 and 1:10 using 96-well round-bottom plates and acquired for analysis.

### Proliferation assay

CD4^+^ T cells were isolated by MACS (manual cell separator) and stained with a cell division tracking dye CFSE at 5μM concentration for 4 min. Then equal volume of fetal bovine serum (FBS) was added to stop the reaction. The cells were resuspended in warm RPMI 1640 medium with 10% FBS and seeded into anti-CD3/CD28 Abs-coated plates for 72 h and 96 h incubation in different concentrations of MLN4924. After stained with FITC-CD4 antibody, flow cytometry was employed for the measurement of proliferation.

### Apoptosis assay

Flow cytometry and Annexin V-FITC Apoptosis Detection Kit I (556547, BD Bioscience) were employed for apoptosis analysis according to the manufacturer's introduction. Cells of each group were stained with Annexin V and then evaluated by flow cytometry.

### Cell viability analysis

Trypan blue exclusion test was employed for detecting cell viability. After different treatments, BMDCs were harvested and counted. Average percentage of Trypan Blue positives and negative cells were calculated in 5-7 randomly selected fields. Trypan Blue negative cells were regarded as living cells.

### Quantification of caspase activity

Cells were resuspended in lysis buffer and incubated on ice for 15 min following the MLN4924 treatment. Caspase-3 activity was determined based on the ability of caspase-3 to change acetyl-Asp-Glu-Val-Asp *p*-nitroanilide (Ac-DEVD-*p*NA) into *p*-nitroaniline (*p*NA), a yellow product. Assays were performed on 96-well plates by incubating 50μl protein lysate per sample in 40μl reaction buffer containing 10μl caspase-3 substrate using caspase activity kit (Beyotime Institute of Biotechnology). After 2 hours incubation in 37°C, the release of *p*NA catalyzed by caspases-3 was quantified by determining absorbance at 405 nm. Caspase-3 activity was expressed as the fold of enzyme activity compared to that of synchronized cells.

### Purification of DCs

Dendritic cells were cultured as described before. On day 6, cells were purified using CD11c microbeads (Meltenyi biotech) according to the manufacturer's instructions. The CD11c^+^ cells were rested for 40 min and whole cell lysates were acquired for immunoblotting after LPS stimulation.

### Immunoblotting

For immunoblotting assay, cell pellets were lysed with RIPA in the presence of Protease Inhibitor Cocktail (Pierce, USA) and protein concentration was determined by Bradford assay. Cell lysates were resolved by SDS/PAGE and immunoblotted with the appropriate antibodies according to the standard protocols. After corresponding HRP-conjugated antibodies incubation, purpose bands were visualized by enhanced chemiluminescence (Pierce). Characteristically, Cul-1 appears as a doublet of 85kD Cul-1 and 90kD Nedd8 conjugated Cul-1. Cul-1 neddylation was reflected as an increase in Nedd8-associated band shift upward in the immunoblots. Signal intensity was analyzed by Image Lab™ Software Version 4.0.1 (BIO-RAD, Hercules, CA).

### Statistical analysis

For statistical tests, Prism 5.0 (GraphPad Soft-ware, SanDiego, CA, USA) was used. Data was presented as means ± SEM from three independent experiments. Data of colon length, weight percentage, clinical score and serum cytokine between mice treated with MLN4924 and control were compared by using the Student's t test. Cytokine secretion, caspase activity, cell viability and protein expression level results among groups receiving different treatments were evaluated with one-way analysis of variance (ANOVA). Statistically significant differences were reported as *p < 0.05, **p < 0.01 and ***p < 0.001.
