# Correction: Inhibition of neddylation regulates dendritic cell functions *via* Deptor accumulation driven mTOR inactivation

**DOI:** 10.18632/oncotarget.26504

**Published:** 2018-12-18

**Authors:** Mengmeng Cheng, Shurong Hu, Zhengting Wang, Yaofei Pei, Rong Fan, Xiqiang Liu, Lei Wang, Jie Zhou, Sichang Zheng, Tianyu Zhang, Yun Lin, Maochen Zhang, Ran Tao, Jie Zhong

**Affiliations:** ^1^ Department of Gastroenterology, Ruijin Hospital, Shanghai Jiao Tong University School of Medicine, Shanghai, China; ^2^ Department of Surgery, Ruijin Hospital, Shanghai Jiao Tong University School of Medicine, Shanghai, China; ^3^ Department of Hepatobiliary-Pancreatic Surgery, Zhejiang Provincial People’s Hospital, Hangzhou, China

**This article has been corrected:** The correct Grant support information is given below:

**GRANT SUPPORT**

We thank Tonghui Ma for his technical help and Xinyu Liu for helpful discussions. This work was supported by Science & Technology department of Shanghai (13411950901), Zhejiang Provincial Nature and Science Foundation (NSF) grant for “Outstanding Youth”(No.LR15H100001 to RT).

Original article: Oncotarget. 2016; 7:35643-35654. https://doi.org/10.18632/oncotarget.9543

